# Construction of S-Scheme BiVO_4_/Bi_2_O_2_S Heterojunction for Highly Effective Photocatalysis of Antibiotic Pollutants

**DOI:** 10.3390/molecules31010136

**Published:** 2025-12-30

**Authors:** Dongdong Chen, Siting Hu, Zhenzhen Jia, Yang Zhang, Bo Zhang, Shasha Liu, Xiang Li

**Affiliations:** Guangdong Provincial Key Laboratory of Environmental Health and Land Resource, School of Environmental and Chemical Engineering, Zhaoqing University, Zhaoqing 526061, China; 18025476768@163.com (S.H.); zhangyang@zqu.edu.cn (Y.Z.); david_zhang200309@163.com (B.Z.); liushasha@zqu.edu.cn (S.L.); lixiang@zqu.edu.cn (X.L.)

**Keywords:** TC photodegradation, BiVO_4_/Bi_2_O_2_S, S-scheme heterojunction, charge separation

## Abstract

Photocatalytic processes have emerged as an efficacious strategy for the removal of organic pollutants from wastewater. In the present investigation, a BiVO_4_ nanorod supported on Bi_2_O_2_S nanosheet catalyst (referred to as BiVO_4_/Bi_2_O_2_S) was meticulously synthesized via a straightforward synthetic approach, aimed explicitly at the photodegradation of tetracycline (TC). The optimized BiVO_4_/Bi_2_O_2_S composite, with a theoretical weight ratio of BiVO_4_ to Bi_2_O_2_S at 2:1 (designated as 2BVO/BOS), demonstrated a significant improvement in tetracycline degradation efficiency, achieving up to 82.9% under visible light irradiation for 90 min. This result stands in stark contrast to the relatively low degradation rates of 42.9% and 50.7% observed for pure BiVO_4_ and Bi_2_O_2_S, respectively. Furthermore, the apparent reaction rate of 2BVO/BOS (approximately 0.01894 min^−1^) was 3.19-fold and 2.66-fold higher than those of BiVO_4_ (0.00594 min^−1^) and Bi_2_O_2_S (0.00713 min^−1^), respectively. This significant improvement in photocatalytic efficacy can be ascribed to the composite’s superior capacity for visible light absorption, as well as its remarkable proficiency in charge carrier separation and transfer. Comprehensive experimental analyses, corroborated by extensive characterization techniques, revealed the formation of a distinctive S-scheme charge transfer mechanism at the interface between BiVO_4_ and Bi_2_O_2_S. This mechanism effectively suppresses charge recombination and optimizes the redox potentials of the photogenerated carriers, thereby enhancing the overall photocatalytic performance. The current study underscores the remarkable potential and promising application of BiVO_4_/Bi_2_O_2_S composite in the realm of wastewater treatment.

## 1. Introduction

The advent of antibiotics has significantly reduced mortality rates over the past few decades, positioning them as some of the most crucial medications for combating bacterial infections. Tetracyclines (TCs), including TC and its derivatives (such as oxytetracycline, tigecycline, and so on), are broad-spectrum antibiotics extensively used to combat Gram-negative and Gram-positive bacteria in both human and animal healthcare [1,2,3,4,5]. Over the past several decades, the overuse and uncontrolled discharge of TCs have not only posed a threat to human health but have also contributed to severe environmental contamination. As such, the development of green, sustainable, and efficient methods for TC removal has become an urgent priority. In response, various advanced treatment technologies, including photocatalysis [6,7,8], electrocatalysis [9], and ozonolysis [10], have been explored for the effective elimination of such organic contaminants from aqueous environments. Among these, photocatalysis stands out as a potent method for mineralization and photodegradation.

Bismuth-based semiconductors, including BiVO_4_, Bi_2_WO_6_, Bi_2_O_2_CO_3_, Bi_2_O_3_, BiOCl, and their analogs, have attracted substantial scholarly interest due to their exceptional photocatalytic capabilities, particularly in the context of TC degradation. This impressive performance is attributed to their expansive energy bandgaps (E_g_), which range from 1.8 to 3.3 eV [11,12]. Furthermore, these materials demonstrate superior visible light absorption, a feature stemming from the hybridized valence band composed of O 2p and Bi 6s orbitals, which effectively narrows the E_g_ values [12]. Among them, monoclinic bismuth vanadate (BiVO_4_), with an E_g_ of about 2.40 eV, is heralded as one of the most promising photocatalysts, owing to its robust physicochemical stability, tunable morphology, and its environmentally friendly, cost-effective nature [13,14,15,16]. Despite these advantageous properties, BiVO_4_’s actual photoelectric conversion efficiency—hovering around 10%—remains far from adequate to fulfill the stringent demands of industrial applications [17]. The primary impediments to the widespread application of BiVO_4_ in photocatalytic processes include insufficient solar energy utilization and the rapid recombination of photogenerated electron–hole (e^−^-h^+^) pairs, which undermine its overall efficacy [17,18]. To address these challenges, a range of strategies have been proposed to enhance the photocatalytic performance of BiVO_4_, such as morphological engineering [7], heteroatom doping [19], metal deposition [20], crystal plane optimization [21], and the construction of heterojunctions [11,12,22]. Among these, the formation of semiconductor heterojunctions is the most efficacious approach, as it mitigates the inherent limitations of individual materials, significantly augmenting photocatalytic activity by fostering enhanced charge separation [13,14,22]. Over the past few decades, various BiVO_4_-based heterostructures have been developed, including BiVO_4_/BiOI [23], m-BiVO_4_/t-BiVO_4_ [24], BiVO_4_/Bi_6_O_6_(OH)_3_(NO_3_)_3_ [25], and BiVO_4_/Bi_2_S_3_ [26], among others.

The recently identified ultrathin two-dimensional (2D) bismuth oxychalcogenides, specifically the Bi_2_O_2_X (X = S, Se, Te) family, have emerged as a highly promising class of materials owing to their superior carrier mobility, tunable bandgap structures, and exceptional physicochemical stability [27]. Notably, Bi_2_O_2_S, with an energy bandgap ranging from 1.2 to 1.5 eV, exhibits a unique crystal architecture characterized by alternating stacking of [BiO]_2n_^2n+^ and Sn^2n−^ layers, with weak interlayer interactions [28,29]. The intercalation of S^2−^ anions between these cationic layers induces synergistic effects, such as band structure modulation, enhanced interlayer charge transport pathways, and improved structural stability [28,29]. The narrow bandgap of Bi_2_O_2_S facilitates efficient absorption of both visible and near-infrared light, substantially broadening its spectral responsiveness to solar energy [28,29]. Moreover, Bi_2_O_2_S is abundant in raw materials, environmentally benign, simple to synthesize, and non-toxic, making it an ideal candidate for sustainable applications [30]. Consequently, Bi_2_O_2_S has been extensively utilized in the construction of heterojunctions with other semiconductors over the past decades, significantly enhancing the photocatalytic performance of these composite structures. Examples include Ni_2_P/Bi_2_O_2_S [31], Bi_2_O_2_S/Bi_12_TiO_20_ [32], Bi_2_O_2_S/Bi_5_O_7_I/ZA [6], and Bi_2_O_2_S/In_2_O_3_ [33].

In this study, Bi_2_O_2_S nanosheets were synthesized through a straightforward solution-based method, and visible light-driven BiVO_4_/Bi_2_O_2_S composites were fabricated via a hydrothermal strategy. Compared to the pristine BiVO_4_ and individual Bi_2_O_2_S, the BiVO_4_/Bi_2_O_2_S heterostructures exhibited significantly enhanced visible light absorption, reduced charge recombination rates, and superior charge transport capabilities for photoexcited carriers. TC was selected as the typical target pollutant to evaluate the photocatalytic degradation performance of the composites. The BiVO_4_/Bi_2_O_2_S composites demonstrated remarkable efficiency in degrading organic contaminants within 90 min. Furthermore, the underlying degradation mechanism was investigated in detail, with free radical trapping experiments and electron spin resonance (ESR) analysis employed to probe the possible pathways. This research offers valuable insights into the development of highly efficient, cost-effective, and non-precious metal-based photocatalysts for environmental applications.

## 2. Results and Discussion

### 2.1. Characterization of the Photocatalysts

**Figure 1:**
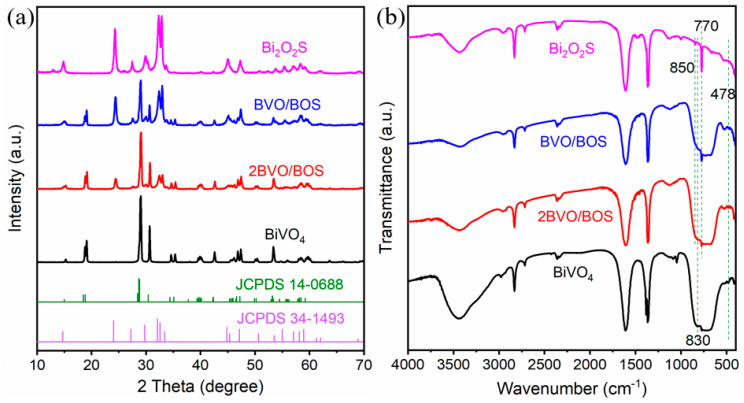
(**a**) XRD and (**b**) FT-IR spectra of the pure BiVO_4_, Bi_2_O_2_S, BVO/BOS, and 2BVO/BOS composites.

Figure 1 presents XRD patterns and FT-IR spectra of BiVO_4_, Bi_2_O_2_S, and their corresponding BiVO_4_/Bi_2_O_2_S composite heterostructures (i.e., 2BVO/BOS and BVO/BOS). In Figure 1a, distinct diffraction peaks are observed at 2θ values of 18.67°, 18.99°, 28.83°, 28.95°, 30.55°, and 53.31°, which can be attributed to the (110), (011), (−121), (121), (040), and (161) crystal planes of the monoclinic BiVO_4_ phase (JCPDS No. 14-0688), respectively [12,34]. In contrast, the XRD pattern of the pristine Bi_2_O_2_S sample reveals peaks at 14.88°, 24.23°, 27.42°, 29.96°, 32.29°, 32.78°, 45.04°, and 55.26°, which are indexed to the (020), (110), (120), (040), (130), (101), (141), and (221) planes of the orthorhombic Bi_2_O_2_S phase (JCPDS No. 34-1493) [30,35,36]. The XRD spectra of the composite materials clearly exhibit characteristic peaks corresponding to both BiVO_4_ and Bi_2_O_2_S, confirming the successful incorporation of both phases within the composite structure. Additionally, as the BiVO_4_ content in the composites decreases, the intensity of the characteristic peaks of Bi_2_O_2_S increases, further substantiating the successful synthesis of the BiVO_4_/Bi_2_O_2_S heterostructures. To elucidate the functional groups inherent in the synthesized catalysts, FT-IR spectroscopy was employed. As depicted in Figure 1b, the spectra of all synthesized composites exhibit absorption bands spanning the wavenumber range of 400–4000 cm^−1^. The pristine BiVO_4_ sample reveals a subtle Bi–O absorption band at 478 cm^−1^, indicative of the bismuth oxide phase [37]. Furthermore, a prominent and expansive absorption peak near 830 cm^−1^ is observed, which corresponds to the bending vibration of the VO_4_^3−^ tetrahedral units, thereby confirming the presence of BiVO_4_ [38]. Additionally, a broad FT-IR band between 3200–3400 cm^−1^ is detected, attributed to the O–H stretching vibration of adsorbed water molecules on the surface of the sample [38]. In the case of pure Bi_2_O_2_S, distinct peaks at approximately 770 and 850 cm^−1^ are observed, which are characteristic of Bi–S bond vibrations [39,40]. The FT-IR spectra of the composite materials unequivocally demonstrate the retention of all functional groups present in the individual BiVO_4_ and Bi_2_O_2_S phases within the synthesized composites. Significantly, an increase in the intensity of the 770 cm^−1^ peak with rising Bi_2_O_2_S content further corroborates the successful incorporation of BiVO_4_ and Bi_2_O_2_S into the composite structure, confirming the formation of a well-integrated heterostructure.

**Figure 2:**
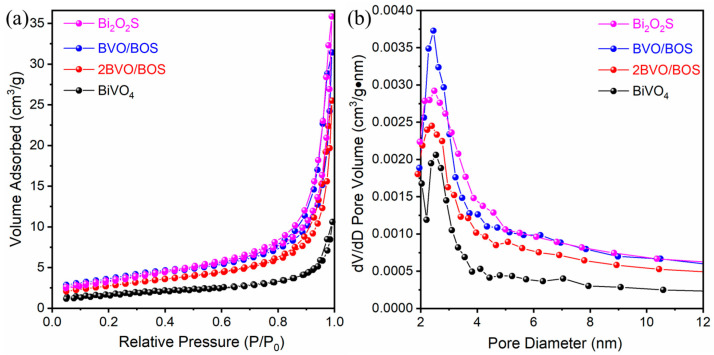
(**a**) N_2_ adsorption–desorption curves; (**b**) the related pore size distribution of the BiVO_4_, Bi_2_O_2_S, BVO/BOS, and 2BVO/BOS composite heterostructures.

To assess the BET-specific surface areas and pore distributions of the catalysts, nitrogen adsorption–desorption isotherms for BiVO_4_, Bi_2_O_2_S, and their BiVO_4_/Bi_2_O_2_S composite counterparts were conducted, with the results presented in Figure 2. The N_2_ adsorption–desorption isotherms, as depicted in Figure 2a, exhibit a characteristic type IV curve with an H3-type hysteresis loop, indicative of a mesoporous structure [37]. The BET surface areas for BiVO_4_, 2BVO/BOS, BVO/BOS, and Bi_2_O_2_S are 6.1, 9.9, 12.1, and 11.9 m^2^/g, respectively. Compared to BiVO_4_, the elevated surface areas of 2BVO/BOS and BVO/BOS can be attributed to the incorporation of Bi_2_O_2_S nanosheets, which significantly enhance the material’s surface properties. As illustrated in Figure 2b, the pore size distribution curves derived from the BJH method, utilizing the adsorption branch of the isotherms, reveal pore sizes predominantly in the range of 2–10 nm for all samples. The incorporation of Bi_2_O_2_S (with a pore volume of 0.0554 cm^3^/g) resulted in a notable increase in pore volume, with values escalating from 0.0164 cm^3^/g for BiVO_4_ to 0.0395 cm^3^/g for 2BVO/BOS and 0.0486 cm^3^/g for BVO/BOS, respectively. The enhanced BET surface area and pore volume of the composites are expected to facilitate photocatalytic reactions by providing an increased number of adsorption and reaction sites, thereby improving overall catalytic efficiency.

**Figure 3:**
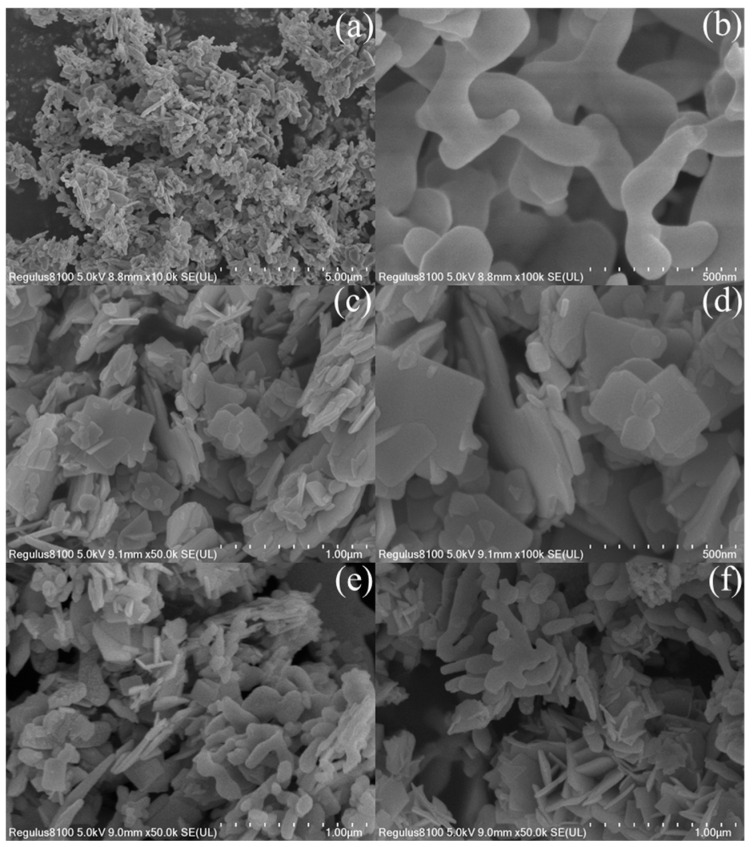
FE-SEM images of BiVO_4_ (**a**,**b**), Bi_2_O_2_S (**c**,**d**), and 2BVO/BOS composite (**e**,**f**) at different magnifications.

**Figure 4:**
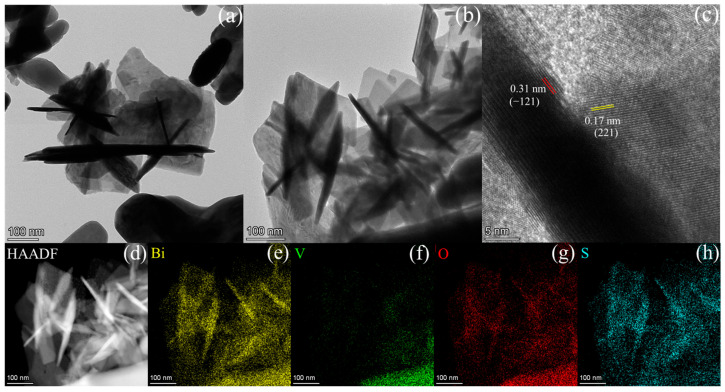
TEM (**a**,**b**), HRTEM images (**c**), and the EDX elemental mapping images (**d**–**h**) of 2BVO/BOS composite (Bi, V, O, and S elements).

To thoroughly investigate the microstructural attributes of the synthesized catalysts, the individual BiVO_4_, Bi_2_O_2_S, and 2BVO/BOS composites were subjected to an array of advanced characterizations using SEM, TEM, and high-resolution TEM (HRTEM), with the corresponding results presented in Figure 3 and Figure 4. As illustrated in Figure 3a,b, the BiVO_4_ sample exhibits a worm-like, nanostructured morphology comprising irregular nanorods. Figure 3c,d reveal that Bi_2_O_2_S adopts a highly uniform nanosheet morphology, with an average lateral size of approximately 200 nm. In the case of the 2BVO/BOS composite (Figure 3e,f), both the irregular nanorods and nanosheets are clearly discernible, further corroborating the presence of both BiVO_4_ and Bi_2_O_2_S phases within the composite. To gain deeper structural insights, TEM and HRTEM analyses were conducted, as shown in Figure 4. Figure 4a,b distinctly reveal the highly homogeneous dispersion of rod-like BiVO_4_ structures, which are meticulously aligned on the surface of Bi_2_O_2_S nanosheets within the 2BVO/BOS composite. The HRTEM image presented in Figure 4c exhibits clear lattice fringes at 0.17 nm, attributed to the (221) plane of Bi_2_O_2_S, and at 0.31 nm, corresponding to the (−121) plane of BiVO_4_, thereby offering definitive proof of the successful formation of the BiVO_4_/Bi_2_O_2_S heterostructure [36,41,42]. Moreover, the high-resolution image in Figure 4c further demonstrates the intimate interface between BiVO_4_ and Bi_2_O_2_S, suggesting a robust interfacial contact that is conducive to enhanced carrier transfer processes. Additionally, the elemental composition and spatial distribution of the constituent elements within the composite were meticulously analyzed through energy dispersive X-ray spectroscopy (EDS) elemental mapping, providing further insight into the uniform distribution and structural integrity of the 2BVO/BOS composite. Figure 4d–h showcase the distribution maps for Bi, V, O, and S, each distinctly color-coded, which confirm the homogeneous dispersion of these elements throughout the 2BVO/BOS composite. Taken together, these findings provide compelling and robust evidence for the successful synthesis, structural coherence, and uniformity of the 2BVO/BOS photocatalyst.

**Figure 5:**
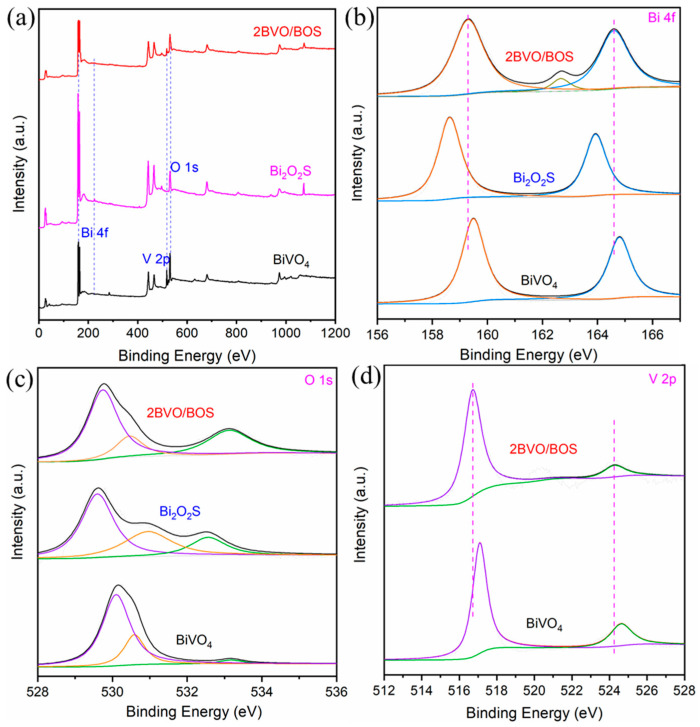
XPS spectra: (**a**) survey spectra, (**b**) Bi 4f, and (**c**) O 1s for the Bi_2_O_2_S, BiVO_4_, and 2BVO/BOS samples; (**d**) V 2p for BiVO_4_ and 2BVO/BOS samples.

To investigate the surface chemical composition and electronic states, XPS analysis was employed to examine both BiVO_4_, Bi_2_O_2_S, and 2BVO/BOS samples. As illustrated in Figure 5a, the survey spectrum indicates that the composite material contains all the elements from the two individual components. The Bi 4f peak illustrated in Figure 5b is characterized by a distinct splitting into the Bi 4f5/2 and Bi 4f7/2 orbitals, with the respective energy positions at 164.8 eV and 159.5 eV for monomeric BiVO_4_, and 163.9 eV and 158.6 eV for Bi_2_O_2_S. The observed spin-orbit splitting energy difference of 5.3 eV signifies the presence of bismuth in the +3 valence state within both BiVO_4_ and Bi_2_O_2_S [29]. In the case of the 2BVO/BOS composite, the Bi 4f5/2 and Bi 4f7/2 orbitals are found at 164.6 eV and 159.3 eV, respectively, indicating positions that lie between those of BiVO_4_ and Bi_2_O_2_S. This intermediate positioning suggests the occurrence of charge transfer between the two materials, highlighting their interaction within the composite structure [13]. In the 2BVO/BOS composite, these binding energies exhibit a minor shift (approximately 0.2 eV) to values of 164.6 eV and 159.3 eV, suggesting a charging effect attributed to the conductive properties of Bi_2_O_2_S [32,43]. The small bump located at 162.7 eV corresponds to the characteristic peak of S [29]. The O 1s XPS spectra (Figure 5c) reveal three distinct peaks located at approximately 530.1 eV, 530.6 eV, and 533.2 eV. The first peak is attributed to the oxygen species present in metal-oxygen bonds, while the latter two peaks correspond to various hydroxyl (OH) groups on the surface; specifically, they represent the dissociative adsorption of water and the irreversibly adsorbed molecular water [44,45,46]. Notably, the binding energy of the O 1s peak is slightly shifted in the 2BVO/BOS composite, further indicating the formation of binding interactions between BiVO_4_ and Bi_2_O_2_S. The significantly weaker intensity of the O 1s peak in the 2BVO/BOS system, relative to pure BiVO_4_, further corroborates the loss of oxygen through the cleavage of Bi–O bonds during the synthesis process [29]. Furthermore, as shown in Figure 5d, the V 2p spectrum of pure BiVO_4_ reveals two characteristic peaks at binding energies of 517.1 eV and 524.6 eV, corresponding to V 2p_3_/_2_ and V 2p_1_/_2_, respectively, with a peak separation of 7.5 eV, confirming the presence of vanadium in the +5 oxidation state (V^5+^) [13]. In the 2BVO/BOS composite, these V 2p peaks shift to lower binding energies relative to the pure BiVO_4_, a phenomenon attributed to the strong interaction between BiVO_4_ and Bi_2_O_2_S [13,47].

**Figure 6:**
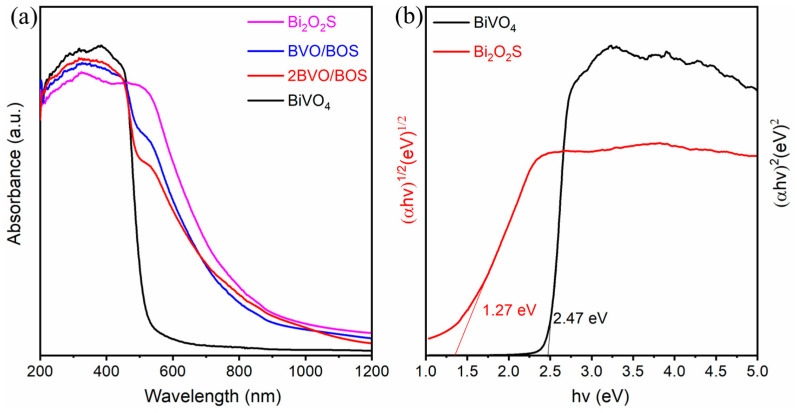
(**a**) UV-Vis spectra of BiVO_4_, Bi_2_O_2_S, BVO/BOS, and 2BVO/BOS composite and (**b**) corresponding Tauc plot for BiVO_4_ and Bi_2_O_2_S.

The light absorption properties of the as-prepared samples are investigated using the UV-Vis DRS method. As shown in Figure 6a, the UV-Vis diffuse reflectance spectra of BiVO_4_, Bi_2_O_2_S, and BiVO_4_/Bi_2_O_2_S composites are obtained. As can be seen, the characteristic absorption edge of pure BiVO_4_ is observed at about 500 nm, while Bi_2_O_2_S exhibits a prominent absorption capability across the entire wavelength range because of its narrow bandgap, showing its remarkable light-trapping structure and photosensitivity. Compared with pure BiVO_4_, the 2BVO/BOS sample exhibited a red-shift spectral response in the visible light region due to a photosensitizing effect of the incorporated Bi_2_O_2_S, with further increasing the Bi_2_O_2_S nanosheet, BVO/BOS shows a stronger ability for adsorbing visible light. The enhanced visible light adsorption ability of the composites means that more electron–hole pairs can be produced under the same visible light irradiation with the assistance of Bi_2_O_2_S. The bandgap of a semiconductor can be calculated by the following formula: αhν = A(hν − E_g_)^n^, where α is the absorption coefficient, h is Planck’s constant, ν is the light frequency, A is a constant, E_g_ is the bandgap energy of the semiconductor, and n depends on the type of transition. The parameter n is determined by the semiconductor’s electronic transition characteristics, with n = 1/2 for direct transitions and n = 2 for indirect transitions [32,48]. Since BiVO_4_ and Bi_2_O_2_S are indirect and direct semiconductors, the E_g_ values of BiVO_4_ and Bi_2_O_2_S can be calculated to be 2.47 eV and 1.27 eV, respectively, by using the Tauc plot analysis method, as depicted in Figure 6b.

**Figure 7:**
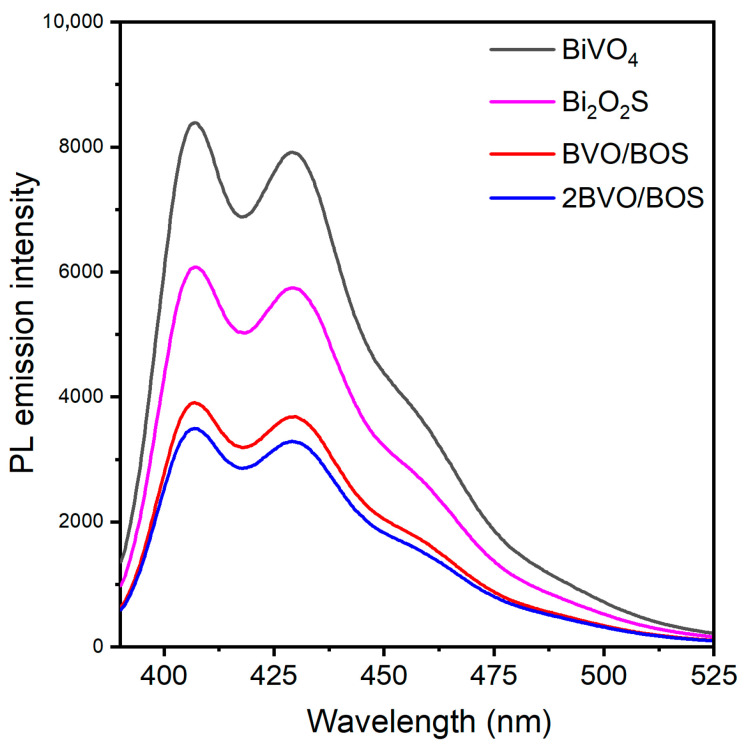
PL spectra of BiVO_4_, Bi_2_O_2_S, BVO/BOS, and 2BVO/BOS composite.

**Figure 8:**
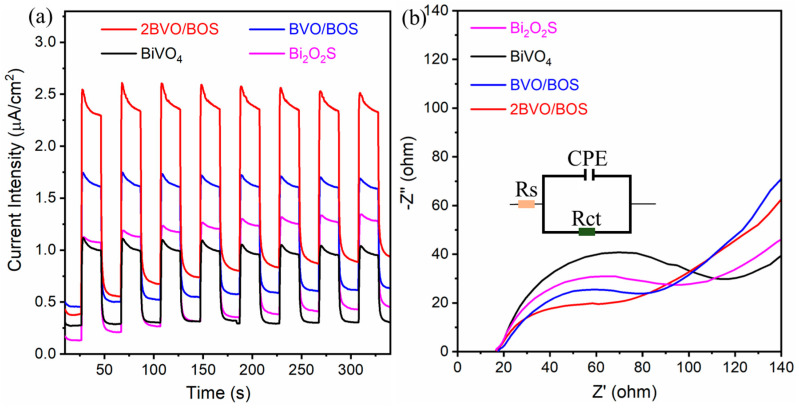
(**a**) Transient photocurrent response and (**b**) EIS spectroscopy of BiVO_4_, Bi_2_O_2_S, BVO/BOS, and 2BVO/BOS composite.

PL spectroscopy stands as an indispensable technique for probing the recombination dynamics of photoinduced electron–hole pairs, a pivotal determinant in the modulation of photocatalytic efficacy. The analysis was executed at an excitation wavelength of 380 nm. The resultant PL spectra of BiVO_4_, Bi_2_O_2_S, BVO/BOS, and 2BVO/BOS are illustrated in Figure 7. Upon the integration of BiVO_4_ with Bi_2_O_2_S, a pronounced diminution in PL intensity was detected, with 2BVO/BOS manifesting the most attenuated emission. The intensity hierarchy of the PL spectra for the synthesized samples follows the sequence: BiVO_4_ > Bi_2_O_2_S > BVO/BOS > 2BVO/BOS, signifying that the 2BVO/BOS composite excels in facilitating the most efficient segregation of photo-excited electron–hole pairs, thus underpinning its enhanced photocatalytic performance [8,32]. These PL observations corroborate the photocatalytic degradation activity for TC, as corroborated by subsequent photocatalytic assays. To further elucidate the separation and transport characteristics of the photogenerated charge carriers, transient photocurrent response (i-t) measurements and electrochemical impedance spectroscopy (EIS) analyses were conducted on the as-prepared BiVO_4_, Bi_2_O_2_S, BVO/BOS, and 2BVO/BOS samples, as shown in Figure 8. The i-t plots depicted in Figure 8a demonstrate that the photocurrent densities of BVO/BOS and 2BVO/BOS surpass those of BiVO_4_ and Bi_2_O_2_S under visible light irradiation. Notably, 2BVO/BOS exhibits the highest current intensity, indicating that the incorporation of Bi_2_O_2_S substantially enhances the migration and separation of photogenerated electron-hole pairs, thereby inducing a more pronounced photocurrent response. EIS is employed to evaluate the electrochemical resistance of the electrode material and the interfacial interactions between the electrode and the electrolyte. The impedance spectra typically exhibit three distinct components across the frequency spectrum: ohmic resistance (Rs), charge transfer resistance (Rct), and a constant phase element (CPE) that accounts for non-ideal capacitive behavior [6,49,50]. Figure 8b showcases the EIS data for the four samples. In the low-frequency region, the diffusion impedance manifests as a well-defined semicircular arc, indicative of the diffusion of species through the electrode films and the presence of direct current flow within the films. It is well-established that a lower Rct value correlates with more rapid charge transfer across the electrolyte/electrode interface [51]. Consequently, a reduced Nyquist semicircle radius in the EIS spectra signals a lower interfacial charge transfer resistance, suggesting superior charge transfer capabilities. The smallest arc radius observed for the 2BVO/BOS composite correlates with the lowest resistance to carrier diffusion, which aligns seamlessly with the trend observed in photocurrent density.

### 2.2. Photocatalytic Degradation of TC

**Figure 9:**
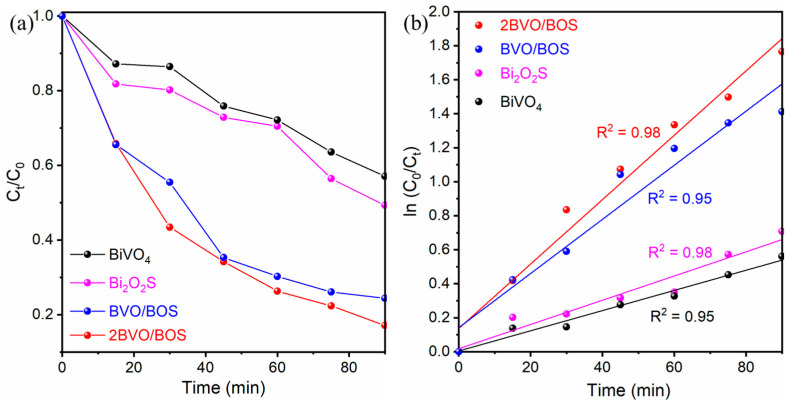
(**a**) The photocatalytic performances for TC degradation under the visible irradiation and (**b**) The pseudo-first-order reaction kinetics of the BiVO_4_, Bi_2_O_2_S, BVO/BOS, and 2BVO/BOS heterostructures.

TC is employed as a benchmark probe to assess the photocatalytic performance of the four as-prepared catalysts under visible light exposure. Prior to illumination, a 90 min dark adsorption step is performed to achieve adsorption–desorption equilibrium within the reaction system. The photocatalytic degradation of TC under visible light for the synthesized samples is presented in Figure 9, with the self-degradation of pure TC being negligible and insignificant. As illustrated in Figure 9a, the bare BiVO_4_ and Bi_2_O_2_S catalysts demonstrated relatively modest degradation rates of 42.9% and 50.7%, respectively, after 90 min of visible light irradiation. This can be attributed to the rapid recombination of photoexcited charge carriers. In stark contrast, the 2BVO/BOS heterostructure exhibited notably superior photocatalytic efficiency, achieving degradation rates of approximately 82.9%, surpassing the individual BiVO_4_ and Bi_2_O_2_S catalysts. The enhanced photocatalytic efficacy of the BiVO_4_/Bi_2_O_2_S composite is ascribed to its superior charge carrier separation and its augmented light absorption in the visible spectrum. To gain further insights into the degradation kinetics of TC, the photocatalytic performance of the materials was analyzed through a first-order kinetic model. As shown in Figure 9b, the rate constants for BiVO_4_, Bi_2_O_2_S, BVO/BOS, and 2BVO/BOS heterostructures were determined from the linear plot of ln(C_0_/C_t_) versus reaction time. The linearity of this plot unequivocally confirms that TC degradation follows a pseudo-first-order kinetic model [51]. The degradation process for all samples adheres to the pseudo-first-order kinetic equation: ln(C_0_/C_t_) = kt, where k represents the rate constant, t denotes the irradiation time (in minutes), and C_0_ and C_t_ are the initial and subsequent concentrations of TC, respectively [50,51]. From this model, the apparent rate constants for the photocatalysts were extracted. As depicted in Figure 9b, the 2BVO/BOS composite exhibits the highest apparent rate constant of 0.01894 min^−1^, significantly surpassing the rate constants of BiVO_4_ (0.00594 min^−1^), Bi_2_O_2_S (0.00713 min^−1^), and BVO/BOS (0.01592 min^−1^). Remarkably, the 2BVO/BOS composite exhibits an apparent rate constant that is approximately 3.19, 2.66, and 1.19 times greater than those of BiVO_4_, Bi_2_O_2_S, and BVO/BOS, respectively. This substantial enhancement in photocatalytic activity provides compelling evidence of the synergistic effects arising from the integration of BiVO_4_ and Bi_2_O_2_S within the heterostructure, underscoring the exceptional charge transfer dynamics and enhanced photocatalytic efficiency of the BiVO_4_/Bi_2_O_2_S composite.

**Figure 10:**
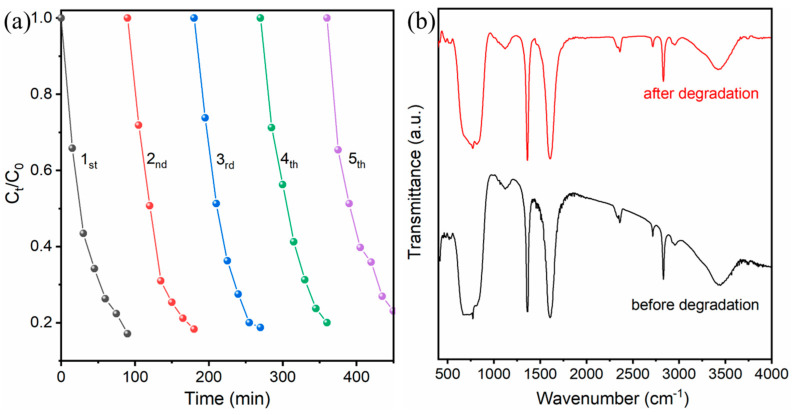
(**a**) Five cycling experiments of 2BVO/BOS heterostructure for photocatalytic TC degradation; (**b**) FT-IR spectra of 2BVO/BOS heterostructure before and after TC degradation.

Stability constitutes a pivotal criterion in defining the practicality and applicability of an efficient photocatalyst, particularly within the demanding context of wastewater purification [50,52]. Therefore, a rigorous evaluation of the recyclability and long-term structural robustness of the 2BVO/BOS heterostructure is indispensable. To systematically assess its durability, five successive photocatalytic degradation cycles of TC under visible light irradiation were conducted, with the corresponding results depicted in Figure 10a. Remarkably, relative to the initial cycle, the photocatalyst exhibited only a marginal decline—approximately 5%—in degradation efficiency by the fifth run. This minimal attenuation in performance is plausibly attributed to slight material attrition or partial detachment of active sites induced by repeated irradiation and recovery processes. Additionally, FT-IR spectroscopy was employed to analyze the surface chemical composition of the samples (Figure 10b). The results revealed that the photocatalyst retains a consistent chemical composition throughout the photocatalytic cycles, showing no evidence of impurity surface groups before or after the photocatalytic reaction. Collectively, these findings conclusively demonstrate that the 2BVO/BOS heterostructure possesses exceptional physicochemical stability and commendable recyclability, retaining robust photocatalytic performance over multiple operational cycles. Such steadfast durability underscores its formidable potential for sustainable, long-term deployment in environmental remediation systems, affirming its reliability and resilience under prolonged functional conditions.

**Figure 11:**
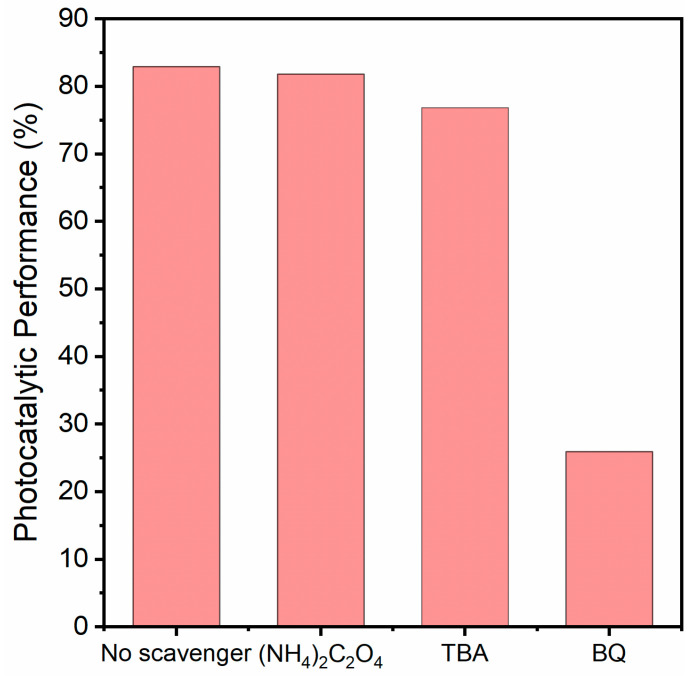
The photocatalytic performance of 2BVO/BOS composite for TC degradation in the presence of various trapping agents.

**Figure 12:**
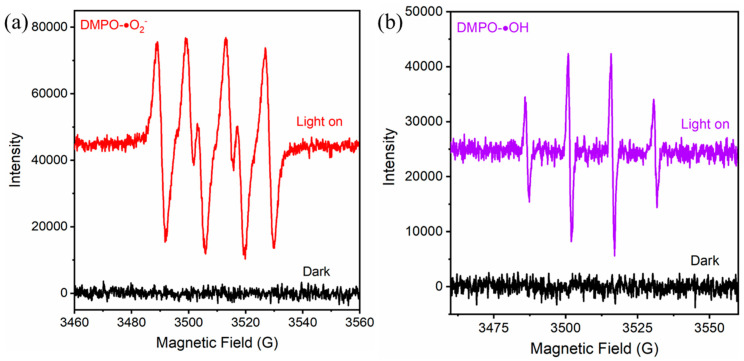
ESR spectra of (**a**) DMPO-•O_2_^−^ and (**b**) DMPO-•OH adducts for 2BVO/BOS in methanol dispersions, recorded both in the dark and under visible light (λ > 420 nm).

To gain deeper insight into the primary reactive species responsible for the photodegradation of TC by the 2BVO/BOS nanocomposite, an exhaustive series of active species scavenging experiments was meticulously carried out. Ammonium oxalate ((NH_4_)_2_C_2_O_4_), benzoquinone (BQ), and tert-butyl alcohol (TBA) were strategically employed as scavengers to selectively capture h^+^, •O_2_^−^, and •OH species, respectively [27,53]. As illustrated in Figure 11, the degradation efficiency of TC was observed to be 82.9% in the absence of any scavenger, establishing the baseline photocatalytic activity. Notably, the introduction of (NH_4_)_2_C_2_O_4_ exerted no discernible effect on the TC degradation efficiency, thereby indicating that h^+^ does not play a predominant role in the degradation process. In contrast, the addition of BQ and TBA led to a noticeable decline in degradation efficiency, thereby providing compelling evidence of the involvement of both •O_2_^−^ and •OH in the photocatalytic process. Specifically, BQ and TBA reduced the photocatalytic performance to 25.9% and 76.8%, respectively, signifying that •O_2_^−^ contributes substantially to the degradation of TC, while •OH plays a secondary, yet significant, role. To further corroborate the participation of •O_2_^−^ and •OH, ESR spectroscopy was employed. As shown in Figure 12a,b, no signals were detected under dark conditions, confirming the absence of reactive species in the absence of visible light illumination. However, upon visible light irradiation of the 2BVO/BOS nanocomposite, distinct ESR signals with characteristic intensity ratios of 1:1:1:1 and 1:2:2:1, corresponding to DMPO-•O_2_^−^ and DMPO-•OH adducts, were clearly observed [6,54]. These results unambiguously confirm the presence of •O_2_^−^ and •OH under light irradiation. The ESR data are in excellent agreement with the scavenging experiment outcomes, further reinforcing the notion that •O_2_^−^ and •OH are pivotal contributors to the enhanced photocatalytic performance of the 2BVO/BOS nanocomposites.

### 2.3. Photocatalytic Mechanism

**Figure 13:**
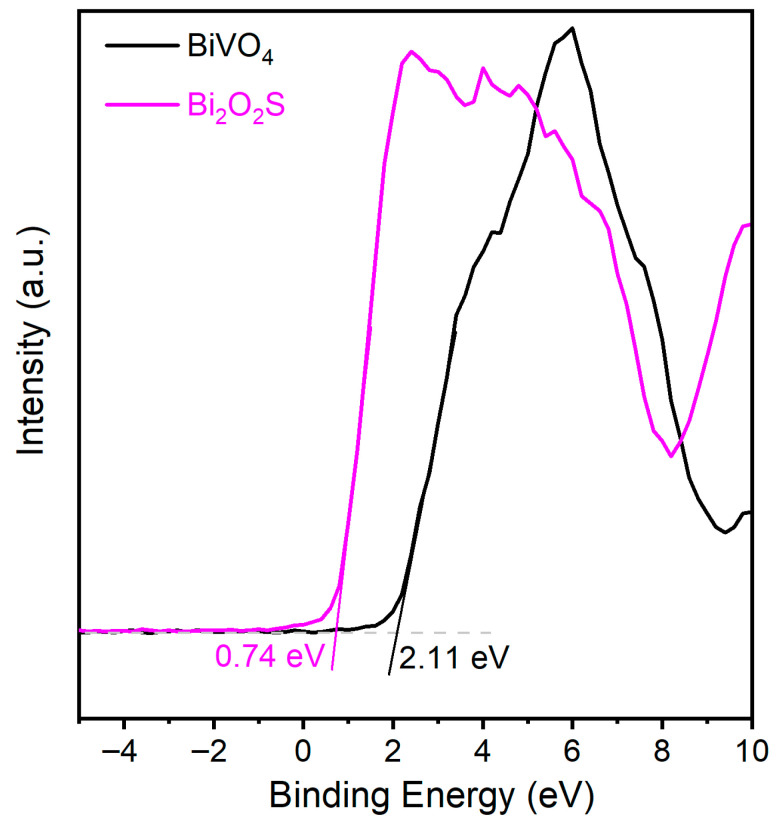
The valence band positions of BiVO_4_ and Bi_2_O_2_S photocatalysts determined by the VB-XPS method.

**Figure 14:**
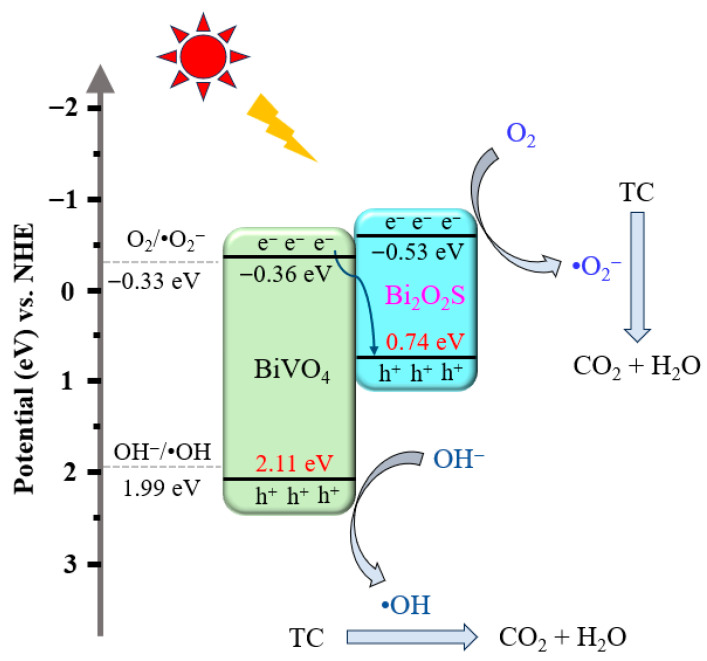
Possible S-scheme heterojunction photocatalytic reaction mechanism for the BiVO_4_/Bi_2_O_2_S composite.

To further elucidate the mechanism underlying the substantial enhancement in the TC degradation performance of the 2BVO/BOS photocatalyst, the positions of the conduction band (CB) and valence band (VB) of the BiVO_4_ and Bi_2_O_2_S samples were determined via valence band X-ray photoelectron spectroscopy (VB-XPS), thereby facilitating an in-depth analysis of the charge transfer dynamics. Specifically, as shown in Figure 13, the valence band positions of BiVO_4_ and Bi_2_O_2_S were ascertained to be 2.11 and 0.74 eV, respectively. Additionally, the optical bandgaps of BiVO_4_ and Bi_2_O_2_S, calculated from the UV-Vis diffuse reflectance spectra (Figure 6b), were found to be 2.47 eV and 1.27 eV, respectively. Consequently, the conduction band positions of BiVO_4_ and Bi_2_O_2_S were calculated to be −0.36 eV and −0.53 eV, respectively. Incorporating the results from the trapping experiments, ESR spectra, redox potential assessments, and charge transfer analysis, a plausible S-scheme type photocatalytic mechanism can be proposed, as illustrated in Figure 14. Under visible light irradiation, the composite absorbs photon energy, leading to the generation of electron-hole pairs that migrate from the VB to the CB. Owing to the energy disparity between the CB and VB positions, the photogenerated electrons and holes undergo interband transfer. Specifically, the excited electrons are transferred from the CB of Bi_2_O_2_S to the CB of BiVO_4_, while the photoexcited holes migrate from the VB of BiVO_4_ to the VB of Bi_2_O_2_S. Considering that the VB position of Bi_2_O_2_S (+0.74 eV) is more negative than the oxidation potential of OH^−^/•OH (+1.99 eV vs. NHE) [13,55], the thermodynamic conditions preclude the formation of •OH radicals. Nevertheless, both •O_2_^−^ and •OH radicals were observed in ESR spectra and radical trapping experiments, confirming their crucial roles in the photocatalytic degradation of TC. Therefore, a more plausible S-scheme type photocatalytic mechanism is proposed, as illustrated in Figure 14. In this mechanism, the excited electrons in the CB of BiVO_4_ are transferred to the interface and recombine with the holes in the VB of Bi_2_O_2_S. Consequently, the electrons remain in the CB of Bi_2_O_2_S, while the holes are retained in the VB of BiVO_4_. The electrons in the CB of Bi_2_O_2_S possess sufficient reducing power to convert O_2_ into •O_2_^−^, owing to the CB potential of Bi_2_O_2_S (−0.53 eV vs. NHE), which is more negative than the standard potential for O_2_/•O_2_^−^ (−0.33 eV vs. NHE) [42,56]. Simultaneously, the holes in the VB of BiVO_4_ (+2.11 eV vs. NHE) exhibit a strong oxidative potential capable of generating •OH radicals. This mechanism significantly suppresses the recombination of electron-hole pairs, leading to the enhanced generation of •O_2_^−^ and •OH radicals, which, in turn, play dominant roles in the photodegradation of TC.

## 3. Experimental Section

### 3.1. Catalyst Preparation

#### 3.1.1. Preparation of Bi_2_O_2_S Nanosheets

The Bi_2_O_2_S nanosheets were synthesized via a refined, modified procedure, building upon established methodologies [35]. In this process, 100 mg (0.206 mmol) of Bi(NO_3_)_3_·5H_2_O was sonicated in 20 mL of deionized water, resulting in a transparent solution. In a separate vial, 12.7 mg of CH_4_N_2_S was introduced to 1 mg of hydrazine hydrate solution, and subsequently, 120 mg of KOH and 320 mg of NaOH were carefully added to the mixture. The reaction was allowed to proceed overnight, yielding a brown precipitate. The solid product was thoroughly washed with distilled water to remove impurities and then dried overnight in a forced-air oven. The resulting material was characterized as deep brown, exhibiting the distinctive morphology of Bi_2_O_2_S nanosheets.

#### 3.1.2. Preparation of BiVO_4_/Bi_2_O_2_S and BiVO_4_ Catalysts

Following the synthesis of Bi_2_O_2_S nanosheets through a straightforward solution-phase approach at ambient temperature, BiVO_4_/Bi_2_O_2_S composite photocatalysts were fabricated via a hydrothermal method, incorporating varying amounts of Bi_2_O_2_S. The theoretical weight ratios of BiVO_4_ to Bi_2_O_2_S were maintained at 2:1 and 1:1, labeled as 2BVO/BOS and BVO/BOS, respectively. In a typical synthesis procedure, equimolar quantities of Bi(NO_3_)_3_·5H_2_O and NH_4_VO_3_ were dissolved in a 2 mol/L nitric acid solution. Ammonia solution was then introduced to adjust the pH of the resulting mixture to 4. The Bi_2_O_2_S nanosheets were subsequently incorporated into the precursor solution and subjected to ultrasonic treatment to ensure uniform dispersion. This homogeneous solution was transferred to a 100 mL stainless-steel autoclave, lined with PTFE, and subjected to hydrothermal treatment at 150 °C for 6 h. The resulting composite materials were repeatedly washed with deionized water and ethanol, followed by drying at 70 °C for 10 h to yield the BiVO_4_/Bi_2_O_2_S composite photocatalysts. For comparison, BiVO_4_ was synthesized using the same procedure, but without the incorporation of Bi_2_O_2_S.

### 3.2. Catalyst Characterization

The crystallographic framework of the catalysts was meticulously investigated through X-ray diffraction (XRD) analysis, utilizing a Bruker D8-Advance diffractometer (Bruker, Karlsruhe, Germany), powered by a Cu Kα radiation source. The specific surface area (S_BET_) and pore size distribution were precisely determined at 77 K using a Micromeritics ASAP 2020 adsorption–desorption analyzer (Micromeritics, Norcross, GA, USA). To probe the molecular structures and surface chemical compositions, Fourier-transform infrared (FT-IR) spectroscopy (FTIR-8400S, Shimadzu, Kyoto, Japan) and X-ray photoelectron spectroscopy (XPS, ESCALAB 250, Thermo Fisher Scientific, Waltham, MA, USA) were employed, respectively. The optical absorption properties were evaluated through diffuse reflectance spectroscopy (UV-Vis DRS) within the 200–1200 nm range, with BaSO_4_ serving as an inert reference (UV3600IPLUS, Shimadzu, Kyoto, Japan). The morphological and microstructural characteristics of the samples were analyzed using field-emission scanning electron microscopy (FE-SEM, Regulus 8100, Hitachi, Hitachi, Japan) in conjunction with transmission electron microscopy (TEM, FEI Tecnai G2 F20, Hillsboro, OR, USA). Additionally, ESR spectroscopy (JES-FA300, Bruker, Karlsruhe, Germany) was utilized to detect and quantify reactive radical species, with 5, 5-dimethyl-1-pyrroline N-oxide (DMPO) employed as the spin-trapping agent to capture •OH (hydroxyl) and •O^2−^ (superoxide) radicals.

### 3.3. Photoelectrochemical Measurements

Electrochemical evaluations were conducted using a CHI 660D electrochemical workstation (Chenhua, Shanghai, China). A three-electrode setup was employed, wherein a platinum (Pt) wire functioned as the counter electrode, a saturated calomel electrode (SCE) was utilized as the reference electrode, and the catalysts were employed as the working electrode. Photocurrent response measurements were performed in a 0.5 M Na_2_SO_4_ aqueous electrolyte under irradiation from a 300 W xenon (Xe) lamp. To assess the charge transfer dynamics and resistance characteristics of the system, electrochemical impedance spectroscopy (EIS) was conducted in the Na_2_SO_4_ solution. An alternating current (AC) signal with a 10 mV amplitude was applied across a frequency range of 10^5^ Hz to 10^−1^ Hz, facilitating a comprehensive analysis of the electrochemical behavior of the material.

### 3.4. Photocatalytic Tests

The photocatalytic performance of the composite materials was evaluated in a photoreactor equipped with a 420 nm cutoff filter and a 300 W xenon lamp (PLS-SXE 300BF, Merry Change, Beijing, China), simulating visible light irradiation. A 50 mL solution of 10 mg/L TC was prepared, to which 50 mg of the synthesized composite materials was added. After a 90 min dark reaction, the lamp was turned on to initiate the photocatalytic reaction. During the reaction, 3 mL of the solution was sampled at 15 min intervals, filtered through a 0.22 μm microporous membrane to remove catalyst particles, and the absorbance at the characteristic absorption wavelength of TC (357 nm) was measured using a UV-Vis spectrophotometer. To comprehensively assess the catalytic performance, the entire photocatalytic degradation experiment was conducted for 90 min, with samples collected at six different time points for analysis. The degradation efficiency of tetracycline is calculated based on the concentration of the tetracycline solution after 1.5 h of dark reaction as the initial benchmark. During the cyclic experiments, after each reaction, the catalyst in the solution is recovered by centrifugation and dried. In each cycle of the reaction, the lost catalyst is supplemented with fresh catalyst. Additionally, each time a new tetracycline solution at a concentration of 10 mg/L is freshly prepared for the cyclic reactions.

## 4. Conclusions

In summary, we delineated a sophisticated and highly effective strategy for constructing a BiVO_4_/Bi_2_O_2_S heterostructure that exhibits outstanding photocatalytic prowess toward TC degradation. The strategic incorporation of Bi_2_O_2_S not only substantially augments the visible light harvesting capability but also markedly accelerates the spatial separation and interfacial migration of photogenerated charge carriers within the composite. In addition, Bi_2_O_2_S modification significantly amplifies the S_BET_ of the composite, thereby providing a considerably greater density of adsorption sites and catalytically active centers. Consequently, the 2BVO/BOS heterostructure delivers an extraordinary photocatalytic performance, achieving an impressive TC removal efficiency of approximately 82.9% under visible light illumination for 90 min—far surpassing those of pristine BiVO_4_ (42.9%) and Bi_2_O_2_S (50.7%). Complementary radical quenching assays and ESR analyses reveal that •O_2_^−^ serves as the dominant oxidative species during the degradation process, while •OH plays an auxiliary yet non-negligible role. On the basis of these mechanistic insights, an S-scheme heterojunction charge-transfer pathway is proposed to rationalize the superior photocatalytic behavior of the 2BVO/BOS system. Collectively, this work establishes an elegant and practical paradigm for engineering next-generation BiVO_4_-based photocatalysts with elevated activity, offering substantial promise for advanced environmental purification technologies.

## Data Availability

The data presented in this study are available upon request from the corresponding author.
